# MedTalks: developing teaching abilities and experience in undergraduate medical students

**DOI:** 10.1080/10872981.2016.1264149

**Published:** 2016-12-16

**Authors:** Suhair Bandeali, Albert Chiang, Christopher J. Ramnanan

**Affiliations:** ^a^ Faculty of Medicine, University of Ottawa, Ottawa, Canada; ^b^ Department of Innovation in Medical Education, Faculty of Medicine, University of Ottawa, Ottawa, Canada

**Keywords:** Medical student, teaching program, teaching skills, teaching experience, feedback, scholar, CanMEDs, students-as-teachers (SAT)

## Abstract

**Objectives:** According to the CanMEDS’ Scholar competency, physicians are expected to facilitate the learning of colleagues, patients and other health professionals. However, most medical students are not provided with formal opportunities to gain teaching experience with objective feedback.

**Methods:** To address this, the University’s Medical Education Interest Group (MEIG) created a pilot teaching program in January 2015 entitled ‘MedTalks’. Four 3-hour sessions were held at the University Faculty of Medicine, where first and second year medical students taught clinically oriented topics to undergraduate university students. Each extracurricular session included three 30-minute content lectures, and a 90-minute small group session on physical examination skills. Each medical student-teacher received formal feedback from undergraduate students and from faculty educators regarding teaching style, communication abilities, and professionalism. In addition, medical student-teachers self-evaluated their own teaching experience.

**Results:** Over 50 medical students from the University participated as medical student-teachers. Based on quantitative and qualitative evaluation surveys, 100% of medical students agreed that MedTalks was a useful way to develop teaching skills and 92% gained a greater confidence in individual teaching capabilities, based largely on the opportunity to gain experience (with feedback) in teaching roles.

**Conclusions:** A program designed to give medical students multi-source teaching experience (lecture- and small group-based) and feedback on their teaching (from learners and Faculty observers, in addition to their own self-reflection) can improve medical student confidence and enthusiasm towards teaching. Future studies will clarify if medical student self-perceived enhancements in teaching ability can be corroborated by independent (Faculty, learner) observations of future teaching activity.

## Introduction

In 2005, the Royal College of Physicians and Surgeons of Canada established seven formal roles to guide the practice of all Canadian physicians. In the role of Scholar, physicians are expected to act as teachers and facilitate the education of their patients, students, fellow healthcare professionals, and the public [1]. Also, several studies have demonstrated numerous benefits for engaging in clinical teaching, including enhanced communication skills and physician-patient interaction, stronger knowledge-base and skill development, and engagement in future formal teaching roles [2–4]. Despite these benefits and College requirements, most Canadian medical schools do not provide formal training opportunities for medical students in educator roles. Moreover, the few medical student teaching programs that are characterized in the literature do not typically offer student-teachers the benefit of objective feedback regarding their performance [4].

Like any other skill, developing teaching acumen requires instruction, practice, and feedback [5]. Several studies have demonstrated an improvement in teaching competence as a result of formal teaching training programs [4,6]. Given that most undergraduate medical students may not have prior formal educator training, it is important to instruct medical students how to educate before they are required to teach others in the clinic. Currently, the training of future physicians as educators is generally limited to post-graduate initiatives such as Resident-as-Teacher (RAT) programs [7]. Compared to their colleagues, residents with prior educator training demonstrate a significant increase in overall teaching effectiveness, especially in terms of knowledge and communication abilities, as well as a greater enthusiasm for teaching [6,7]. Overall, individuals report increased confidence and preparedness to teach in residency after engaging in curricula to formally develop essential teaching skills [4]. However, as teaching is expected as early as the first few months of residency, the development of teaching skills in undergraduate medical students is necessary [2,4]. Developing and applying teaching skills in formal teaching environments are critical training opportunities that are seldom offered at the medical student level.

To address this need, the University Medical Education Interest Group (MEIG) created a pilot teaching program in January 2015 entitled ‘MedTalks’. This novel educational initiative aimed to provide undergraduate medical students with the knowledge and skills to excel in teaching roles during their clinical careers and to provide teaching opportunities with formal observation-based feedback.

The purpose of this report is to characterize the development of the local innovative MedTalks program, and evaluate the program via participant feedback, identifying both strengths and areas requiring improvement. The dissemination of the lessons learned from this study will help further the development of practical teaching programs at the undergraduate medical student level, complete with multi-level feedback.

## Methods

### Components of the program

#### Sessions

MedTalks was held at the University of Ottawa Faculty of Medicine between January 2015 and April 2015, and included four 3-hour evening sessions. Each session included three 30-minute lectures and a 90-minute small group session. These teaching sessions were centered on the anatomy, physiology, pathophysiology, and relevant clinical exam skills pertaining to the four session topics: the cardiovascular system, the respiratory system, the musculoskeletal system, and patient history.

#### Lecture-based teaching

Medical student-teachers, individually or in pairs, delivered a 25-minute lecture, followed by 5 minutes of questioning from learners and open discussion. Lecture materials included: anatomy and physiology teaching slides related to muscles, heart, and lungs; electrocardiogram interpretations; and chest x-ray interpretations. All teaching material was prepared by medical student-teachers using standardized guidelines from the local medical program’s Physician Development Course to ensure quality and consistency of teaching materials.

#### Small-group based teaching

Medical student-teachers demonstrated clinical exam skills to undergraduate student learners in a small group teaching context. Clinical exam skills taught included: auscultation with a stethoscope, use of a sphygmomanometer, palpation, and percussion. Clinical exam skill teaching was based on the local Physician Skills Development course. Students were also brought to the medical gross anatomy lab at the University Faculty of Medicine. Under the supervision of anatomy teaching Faculty, medical student-teachers led student-learners through relevant objectives using prosected cadaveric specimens; these demonstrations were based on the medical curriculum that has been described previously [8].

### Evaluations

#### Medical student-teacher assessment

Two evaluation forms were created to evaluate and provide feedback regarding medical student teaching immediately after each session. These evaluation forms were based on the official undergraduate medical program’s evaluation forms, and included both five-point Likert scale items and gave the option for open-ended qualitative feedback. The first evaluation form (Supplemental File #1) was used by undergraduate learners and faculty evaluators to evaluate the teaching activities, including clarity of lecture material, engagement of audience, and overall effectiveness. The second form (Supplemental File #2) was provided to the medical student-teachers to reflect upon their teaching experience, and comment on any impact this program had on their willingness to participate in future formal (clinical) teaching activities.

While the feedback student-teachers received from Faculty on their lectures were based on Faculty notes from an entire observed lecture, the feedback that student-teachers received from Faculty on small group teaching was limited to short ~5–10 minute observation windows (from the entire 90 minutes session) due to lack of Faculty participants (relative to participating student-teachers and small group sessions) and the consequent need for Faculty to circulate room to room to provide some feedback to all students.

#### MedTalks program evaluation

Undergraduate learners and medical student-teachers also completed program evaluation surveys regarding the MedTalks program that included both quantitative (Likert-scale) and qualitative (open-ended commentary) aspects (Supplemental File #3).

## Results

One hundred and fifteen undergraduate students from the University of Ottawa signed up for MedTalks, and 53 medical students from the University of Ottawa participated in MedTalks as medical student-teachers. Eleven faculty members, each with teaching experience in the local medical curriculum, volunteered their time to observe medical student-teachers during their teaching activities and provide written feedback via provided evaluation forms. The written feedback was collected by MedTalks organizers and provided to the medical student-teachers immediately after their teaching activities were completed.

### Feedback on the MedTalks program

#### Undergraduate student learner feedback

Undergraduate student learner feedback was very positive, with 97% of responding participants either agreeing or strongly agreeing that MedTalks was a useful and interesting program. The organization of MedTalks and professionalism demonstrated by medical-student teachers were also highly regarded. The major theme in the open-ended commentary regarding suggestions to improve the initiative was a greater focus on hands-on learning through the small group sessions and less time dedicated to didactic lectures. A related sub-theme that emerged was greater exposure to the anatomy lab.

#### Medical student-teacher feedback

MedTalks was very well received by medical student-teachers as 53 (100%) of medical student-teachers either agreed or strongly agreed that MedTalks was a useful and interesting way to practice their teaching skills, and would be motivated to teach in the future, in other domains as well as at future MedTalks events. While 92% of the student-teachers found that this experience resulted in a greater confidence with individual teaching capabilities and felt comfortable leading their teaching sessions, preparation and training in teaching was identified as an area of weakness of the program by 2 (4%) of the medical student-teachers.

## Discussion

Teaching is a crucial but widely overlooked skill in the everyday role of the clinician. The Royal College of Physicians and Surgeons in Canada has reaffirmed teaching competency as a necessary skill through the formally defined CanMEDS Roles since 2005, which highlights ‘Clinical Educator’ as a key competency to be achieved by all medical professionals, including trainees. However, despite these formal expectations of clinicians to develop as clinician-educators, few programs are characterized in the literature to be dedicated to teaching skill enhancement at the undergraduate medical education level, and most identified programs lack objective assessment of trainees [9]. Approximately one third of medical student knowledge is attributed to resident teaching [4], which has inspired the implementation of residency teaching skill training programs, including resident-as-teacher (RAT) programs) [7]. However, residents are expected to formally teach medical students as early as in their first year of residency, which underlines the importance of building a foundation of teaching skills starting in medical school [2]. The MedTalks program was designed to address this need for teaching training opportunities in early medical education, as well as to incorporate multisource feedback to facilitate reflection and development of effective clinician-educators.

In its inaugural year of 2015, MedTalks received an overwhelmingly positive response from over 50 medical students interested in teaching, and over 100 undergraduates keen to learn from medical students. MedTalks was regarded as informative and well-organized by both participants and medical student-teachers. In addition, all medical student-teachers agreed that MedTalks was beneficial in providing an avenue to practice teaching and more similar opportunities were needed. Although the amount of prior teaching experiences the cohort had was not noted, most student-teachers found this experience resulted in a greater confidence with individual teaching capabilities. Moreover, all participating student-teachers noted that this program enhanced enthusiasm for future teaching opportunities.

### Teaching and learning theory

There were several improvements identified that could be implemented in future iterations of MedTalks, informed by the feedback to the pilot program. Although medical student-teachers were provided with the hands-on experience, the knowledge and theory behind effective teaching was lacking. MacDougall and Drummond state that although physicians are experts at what they teach, they lack expertise on how to teach effectively and therefore rely on personal experience rather than educational skill [10]. Workshops centered around fundamental education principles should have greater emphasis going forward, so that medical students have a strong evidence-based pedagogical foundation regarding best teaching practices to guide their development as teachers.

### Faculty evaluators and feedback

Feedback is essential in facilitating improvements in an educator’s teaching skills and performance [5]. Unfortunately, the pilot program had relatively limited participation from Faculty members. Therefore, the MedTalks sessions were structured to maximize the ability for committed Faculty to observe and provide feedback for as many student-teachers as possible in a given evening. As a result, feedback of small group teaching was limited to just small windows of time and there was limited capacity for observation-based, personalized feedback, which will need to be addressed in future MedTalks series. In addition, feedback from Faculty members was given directly to medical student-teachers immediately after the MedTalks sessions. Although this ensured timely delivery of feedback, it did not allow analysis of any emerging themes from qualitative commentary provided, nor did it allow for quantification of the data related to the Likert-scale items. Future iterations of the MedTalks program will collect these data, which would be useful in determining whether future teaching performances from the participating medical student teachers are impacted by the quantitative and qualitative feedback.

### Longitudinal assessment: evaluation

In a national survey of student-as-teacher programs in U.S. medical schools, Soriano et al noted the lack of long-term outcome evaluations such as assessment of student-teachers’ teaching competence in residency [11]. Assessing the long-term outcome of early teaching training is an important factor in determining what components of the training are applicable to the clinical setting once medical students progress in their training [11]. Thus to provide a longitudinal assessment of the impact of MedTalks, future sessions will aim to provide multiple teaching opportunities for medical student-teachers over the first two years of medical school, each with specific and timely feedback. Analysis of this feedback will be collected to determine whether teaching performances measurably improve over time, and whether the repeated exposure to teaching experience and feedback is positively perceived by participants. In future iterations of the program, focus groups with semi-structured interview style may be conducted with student-teachers to gain further detailed feedback regarding their experience teaching through MedTalks and its impact on their learning and growth as young medical educators.

## Conclusion

Medical students have little opportunity to hone their teaching skills and receive constructive feedback on their teaching abilities, and yet are expected to teach proficiently as early as their first year of residency. The MedTalks initiative addresses these gaps by providing medical students with teaching opportunities, in practical medical education settings, complete with multi-source feedback. These early positive educational experiences may also prove to build enthusiasm and drive for future roles as medical educators.

## Supplementary Material

Supplementary material
