# Supplementary material for: MedTalks: developing teaching abilities and experience in undergraduate medical students
Source: Med Educ Online. 2016 Dec 16;22(1):1264149. doi: 10.1080/10872981.2016.1264149 (PMC5328353; doi:10.1080/10872981.2016.1264149)
Supplement: Supplementary material [file zmeo_a_1264149_sm5755.zip › 33428-SupplementaryFile_1.pdf]

MEIG Feedback Form: Lectures and Small Group Sessions

Date:________________________

Name of Student Teacher:____

Session Topic (History, MSK, Cardio, Resp):____

Session Type (lecture or small group style):____

Please evaluate your tutor by circling the number that you feel best represents the following statements.

	1 Strongly Disagree	2 Disagree	3 Neutral	4 Agree	5 Strongly Agree
**The tutor was well prepared and familiar with the lecture content**	1	2	3	4	5
**The tutor explained the material clearly and at an appropriate level**	1	2	3	4	5
**The tutor encouraged students to ask questions and gave appropriate answers**	1	2	3	4	5
**The tutor stimulated interest in the material**	1	2	3	4	5
**The tutor showed respect for students**	1	2	3	4	5
**The information was presented in an engaging manner.**	1	2	3	4	5
**Overall, I found my tutor to be an effective teacher**	1	2	3	4	5

What about your student teacher's style of teaching did you find most effective?

What suggestions would you give to help your student teacher improve your learning of the material?

Additional Comments:
