# Supplementary material for: MedTalks: developing teaching abilities and experience in undergraduate medical students
Source: Med Educ Online. 2016 Dec 16;22(1):1264149. doi: 10.1080/10872981.2016.1264149 (PMC5328353; doi:10.1080/10872981.2016.1264149)
Supplement: Supplementary material [file zmeo_a_1264149_sm5755.zip › 33428-SupplemenaryFile_2.pdf]

MEIG Feedback Form: Student Teachers Self-Evaluation

Date:___________________________________

Session Topic (History, MSK, Cardio, Resp):_______________________________________

Session Type (lecture or small group style):_______________________________________

Partner Name (if applicable):____

Please evaluate yourself and MedTalks by circling the number that you feel best represents the following statements.

	1 Strongly Disagree	2 Disagree	3 Neutral	4 Agree	5 Strongly Agree
I managed the teaching time allotted in an effective manner.	1	2	3	4	5
This experience was a useful way and interesting way to practice my teaching skills.	1	2	3	4	5
This experience has motivated me to teach again in the future.	1	2	3	4	5
The experience has increased my confidence level in teaching.	1	2	3	4	5
I would teach at MedTalks again in the future.	1	2	3	4	5
I had appropriate tools to help me conduct my lecture and/or small group sessions.	1	2	3	4	5
MedTalks was well-organized.	1	2	3	4	5
SELF-EVALUATION					
I felt well prepared to teach the lecture and/or small group session.	1	2	3	4	5
I felt comfortable explaining the material in a lecture and/or small group setting.	1	2	3	4	5
I felt comfortable asking students questions and keeping them engaged.	1	2	3	4	5

Please describe any issues you had with the organization of MedTalks (sign-up process, creating teaching material, communication with your leads, etc.)

What did you like best about this teaching experience?

What did you like least about this teaching experience?

One thing you would change about the way you taught the lecture and/or small group session?

What teaching experience do you have prior to MedTalks?

Is formal clinical teaching (ex. medical professor) a consideration for your future career in medicine?

What teaching opportunities would you like MEIG to organize in the future?

What teaching workshops would you be interested in attending? (Please circle Y or N for each)

Learning Styles (Y/N)
Questioning in a clinical setting (Y/N)
Creating effective teaching material (PowerPoints, booklets) (Y/N)
Teaching lectures vs. small groups (Y/N)
Giving feedback (Y/N)

Other teaching workshops you would like MEIG to organize in the future?

What suggestions would you give to help your tutor improve your learning of the material?

Additional comments:
