# Supplementary material for: MedTalks: developing teaching abilities and experience in undergraduate medical students
Source: Med Educ Online. 2016 Dec 16;22(1):1264149. doi: 10.1080/10872981.2016.1264149 (PMC5328353; doi:10.1080/10872981.2016.1264149)
Supplement: Supplementary material [file zmeo_a_1264149_sm5755.zip › 33428-SupplemenaryFile_3.pdf]

MEIG Feedback Form: General Feedback for MedTalks 2015 Series

Date:______________________

Please evaluate MedTalks 2015 by circling the number that you feel best represents the following statements.

	**1 Strongly disagree**	**2 Disagree**	**3 Neutral**	**4 Agree**	**5 Strongly agree**
**MedTalks was run in a well-organized manner.**	1	2	3	4	5
**The students running MedTalks behaved in a respectful and professional manner.**	1	2	3	4	5
**The MedTalks session booklets were available, useful, and facilitated my learning.**	1	2	3	4	5
**The duration of the lectures (30 minutes each for a total of 1.5 hours) was appropriate.**	1	2	3	4	5
**The duration of the small group sessions (1.5 hours) was appropriate.**	1	2	3	4	5

Please list any issues you had with the sign-up process, location, food, or general organization of the session:

	**1 Strongly disagree**	**2 Disagree**	**3 Neutral**	**4 Agree**	**5 Strongly agree**
**The content covered at MedTalks was relevant and practical to my needs and interests.**	1	2	3	4	5

Please list any concepts in this workshop you found difficult and explain why:

Please list any other topics would have liked to be covered:

	1<br>Strongly disagree	2<br>Disagree	3<br>Neutral	4<br>Agree	5<br>Strongly agree
**The amount of time devoted to questions and discussion at each session was satisfactory.**	1	2	3	4	5
**I would recommend MedTalks to others.**	1	2	3	4	5
**Overall, I found MedTalks 2015 useful and interesting.**	1	2	3	4	5

What did you like most about MedTalks?

How could MedTalks be improved?

Any additional comments:
